# Chromatin organization revealed by nanostructure of irradiation induced γH2AX, 53BP1 and Rad51 foci

**DOI:** 10.1038/srep40616

**Published:** 2017-01-17

**Authors:** Judith Reindl, Stefanie Girst, Dietrich W. M. Walsh, Christoph Greubel, Benjamin Schwarz, Christian Siebenwirth, Guido A. Drexler, Anna A. Friedl, Günther Dollinger

**Affiliations:** 1 Angewandte Physik und Messtechnik, Universitaet der Bundeswehr Muenchen, Werner-Heisenberg-Weg 39, 85577 Neubiberg, Germany; 2 Department of Radiation Oncology, Technische Universitaet Muenchen, 81675 Munich, Germany; 3 Department of Radiation Oncology, Ludwig-Maximilians-Universitaet Muenchen, 80336 Munich, Germany

## Abstract

The spatial distribution of DSB repair factors γH2AX, 53BP1 and Rad51 in ionizing radiation induced foci (IRIF) in HeLa cells using super resolution STED nanoscopy after low and high linear energy transfer (LET) irradiation was investigated. 53BP1 and γH2AX form IRIF with same mean size of (540 ± 40) nm after high LET irradiation while the size after low LET irradiation is significantly smaller. The IRIF of both repair factors show nanostructures with partial anti-correlation. These structures are related to domains formed within the chromatin territories marked by γH2AX while 53BP1 is mainly situated in the perichromatin region. The nanostructures have a mean size of (129 ± 6) nm and are found to be irrespective of the applied LET and the labelled damage marker. In contrast, Rad51 shows no nanostructure and a mean size of (143 ± 13) nm independent of LET. Although Rad51 is surrounded by 53BP1 it strongly anti-correlates meaning an exclusion of 53BP1 next to DSB when decision for homologous DSB repair happened.

Ionizing radiation induces a variety of different types of damage when targeted to living cells. Severe damage, which can influence cell survival or lead to carcinogenesis, occurs due to ionizing events in the DNA molecule itself. The most lethal of these types of DNA damages are the double-strand breaks (DSB), as they may lead to genetic alterations which in turn can be responsible for cell death or carcigonesis. Mammalian cells react with a variety of complex response mechanisms to DSB induction. One main reaction is the phosphorylation of the histone variant H2AX at serine 139 (S139) to obtain γH2AX through kinases such as ATM, ATR and DNA-PK^1^. The γH2AX domains occur in mega-base-pair (Mbp) large regions of the chromatin around DSB^2^,^3^,^4^,^5^,^6^ and can be visualized as so-called ionizing radiation induced foci (IRIF)^7^. The recruitment and activation of proteins due to damage induction can later on lead to the repair of DSB. The cell has different repair mechanisms to properly rejoin the ends of a DSB, including the possibly error-prone non-homologous end joining (NHEJ)^8^ and the in most cases error-free homologous recombination (HR)^9^. HR is limited to the S/G2 cell cycle phase, due to the fact that a homologous sister chromatin is needed in close vicinity to the DSB as a template to repair the damaged chromatin^2^,^3^,^4^. As a backup pathway for failed NHEJ in G1 an alternative end-joining pathway (alt-EJ) has previously been identified, which works as a last resort, when the other pathways fail^8^.

Recent work analyzed the clustering of DSB repair factors in detail using high resolution microscopy^10^,^11^,^12^ and nanoscopy^11^,^13^,^14^,^15^,^16^ in combination with state of the art correlation and clustering analysis methods. With these methods it is possible to gain a deeper understanding of the functionality of DSB repair proteins and their interactions. After the first reactions to DSB induction, such as phosphorylation of H2AX (γH2AX), the recruitment of downstream repair proteins starts for NHEJ as well as for HR. One of the key proteins accumulating in Mbp regions around the damage is the mediator protein 53BP1^2^,^3^,^4^, which can in most cell lines be found at all damage regions marked by γH2AX and plays a major role in NHEJ. When end-resection of damaged sites starts, NHEJ is impaired and proteins responsible for HR are recruited. One of the major proteins for HR is Rad51, which builds filamentous structures and links the complementary chromatin strands together^5^,^6^. A recent study also showed that the presence of 53BP1 at the damage site is responsible for the proper function of HR through Rad51^10^. Ochs and co-workers conclude the functional connection through experiments using a knock-down of 53BP1, which resulted in a distinct lack of Rad51 promoted repair and was further verified through colocalization analysis of these two proteins in confocal microscopy. This colocalization at IRIF turned out to be a nanoscopic anticorrelation in the detailed structures, when imaged with better resolution^13^. The relocalization of 53BP1 to the outer parts of the repair regions leaves space for other proteins to interact with the core region of the damage^13^,^15^,^17^,^18^,^19^.

Bekker-Jensen *et al*.^20^ defined the compartment of proteins accumulating at the core region of damage and binding to resected single strands, such as Rad51, as the single-stranded DNA (ssDNA) compartment. Other proteins like γH2AX and 53BP1 binding to outer regions are part of the flanking chromatin compartment. The structure and detailed spatial distribution of the proteins located in these compartments may be linked to the chromatin structure in the cell nuclei and its function. However, a detailed model of the structure and function of the compartments is still missing. One out of several mostly accepted models of higher order chromatin structure is the CT-IC model (CT = chromosome territory, IC = interchromatin compartment)^21^,^22^,^23^,^24^. Here, chromatin is meant to cluster in distinct chromosome territories and the intra- and interchromosomal space is filled by the interchromatin compartment. In between these two regions, a domain called the perichromatin region is located. This border zone is functionally important, as first evidence suggests that it is the location where amongst other processes, transcription, DNA replication and DNA repair occur^25^. This region has a size of 100–200 nm^24^ and is not totally filled with chromatin but offers room for proteins binding to DNA. If the structural size of the flanking chromatin and ssDNA compartment are in the range of 100–200 nm this is an indicator that repair regions follow the structures as described by the CT-IC model. With this it is possible to form a connection between repair compartments and chromatin structure. Here it is shown that such distributions are observable in the structure of IRIF visualized by fluorescence nanoscopy.

It is well known that the number and complexity of DSB are larger when induced by high linear energy transfer (LET) radiation compared to low LET radiation of the same dose^11^,^26^. As a result, high LET radiation leads to higher cell killing^27^ and gene alteration effects^28^,^29^ when compared to the effects at the same dose of low LET radiation. This resulst in a higher relative biological effectiveness (RBE) for these endpoints. Also a recent study gives evidence that there are different structures in the IRIF of high or low LET radiation^11^, which was investigated in this study in detail. Using the improved resolution of the applied STED nanoscopy as compared to the previous study^13^ and utilizing quantitative size and correlation analysis a detailed insight into the structural and functional relationships of DSB repair factors occurring on the nanoscale was obtained.

## Results

Measurements of high LET radiation and the Rad51-53BP1 experiment for low LET proton radiation were performed in 3 different experiments with two repetitions (Table 1) with a number of evaluated IRIF n = 10 for each experiment. The sample size was determined using a confidence interval of 90% and is in detail described in the materials and methods section. The low LET experiment for 53BP1-γH2AX was performed in one repetition with 20 IRIF, which is reasonable to compare to the other results. There the data were pooled as the variation within one experiment was larger than between the experiments.

### IRIF nanostructure

**Figure 1:**
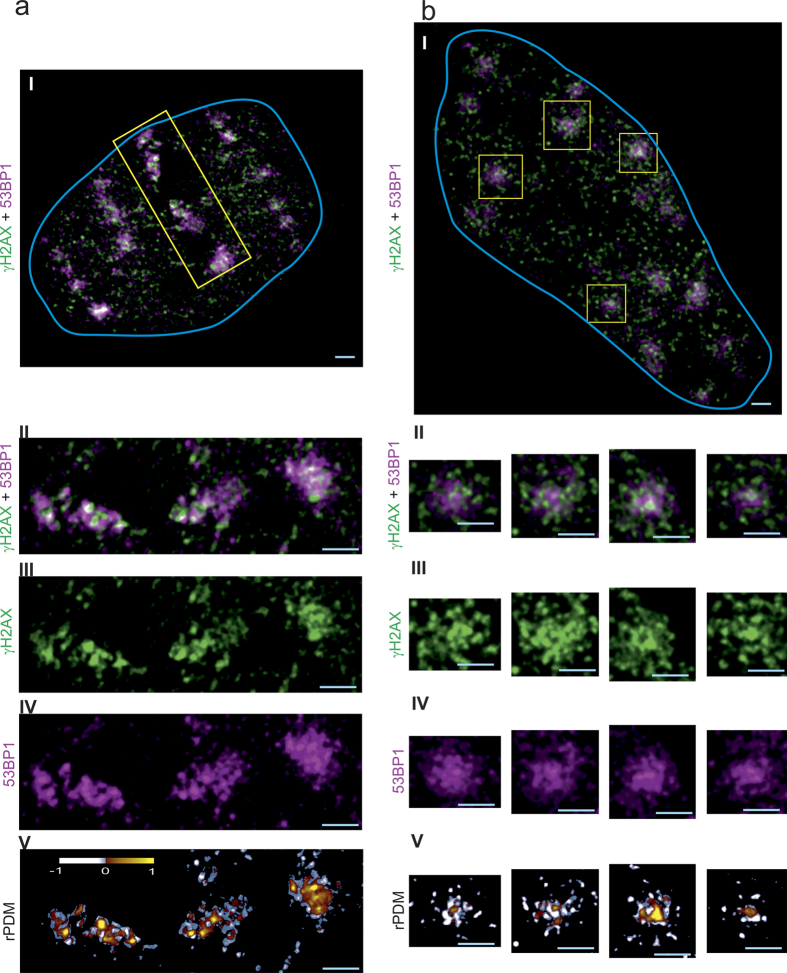
Nanoscopic images of 53BP1 and γH2AX IRIF. In (**a**), a HeLa cell nucleus (blue line) is presented, which was hit by 4 Carbon ions and in (**b**) a nucleus hit by around 200 protons are displayed. 53BP1 (magenta) and γH2AX (green) labelling visualizes the DSB repair regions. Panel I shows the whole cell nuclei, whereas panels II-IV display the enlargement of the yellow-boxed repair regions. For (**a**) one ion track is enlarged and panel II shows the two-color overlay and for (**b**) 4 IRIF are selected and displayed. Magenta, green and white regions are visible in the image, indicating that there is partly correlation between these two repair factors. The γH2AX signal visualized in panel III shows three subcompartments, each consisting of several round or elongated structures with high intensity. The 53BP1 in panel IV appears with similar inner nanostructure indicated by regions with high and regions with low intensity inside the ion track. Panel V shows the rPDM analysis, which proves the partial correlation with the visible yellow and red regions and also the partial anticorrelation, visualized by the blue and white regions. Scale bar: 1 μm.

IRIF induced by irradiation of human HeLa cells with (27 ± 8) MeV carbon ions resulting in an LET value of (500 ± 80) keV/μm and with (20.8 ± 0.1) MeV protons (LET = (2.6 ± 1) keV/μm) were examined. Visualization of IRIF from 53BP1, γH2AX and Rad51 was performed by 3D STED nanoscopy using secondary immunofluorescence staining after 1 h of post-irradiation incubation. This super resolution optical microscopy method provided a resolution of 105 nm in lateral direction and approximately 200 nm in axial direction in this experiment. Figure 1a shows a cell that was hit by 4 carbon ions as it shows 4 parallel string like arrangements of the IRIF for both γH2AX (green) and 53BP1 (magenta). When enlarging the IRIF from one carbon track (yellow marked region) the foci structure displays a non-homogeneous intensity distribution expressing an inner nanostructure. γH2AX manifests in round or elongated nanostructures. The long structures seem to be an interconnection of the round nanostructures, as the small width of the strings is the same as the diameter of the spots. Several of these nanostructures together form single IRIF being distributed along the ionizing track of the carbon ion. For 53BP1 the IRIF are also composed of round or elongated structures similar to that of γH2AX. However, in detail there is only partial overlap leaving many nanoscopic areas where only one of the two factors is visible. In Fig. 1b a cell hit by approximately 200 protons (dose approx. 2 Gy) is shown. Only 15 IRIF are visible as just a single slice of the cell nucleus is shown. The IRIF from proton irradiation are spread over the whole cell nucleus as expected from a random low LET radiation where the induced DSB are produced independently from one another. This random distribution is due to the low ionization density of protons, as an average of 0.2 DSB per proton induced is and therefore 1 DSB per IRIF is expected^13^. The true distribution of the proteins in detail in the cell nucleus is non-random as the proteins accumulate in the perichromatin or low density-chromatin regions. The enlargement of 4 IRIF (yellow boxes in Fig. 1b) shows the same structure for γH2AX as well as for 53BP1 as it was visible in the carbon ion track. Also the correlation analysis shows the same partial correlation for γH2AX and 53BP1 after proton irradiation as after carbon ion irradiation.

**Figure 2:**
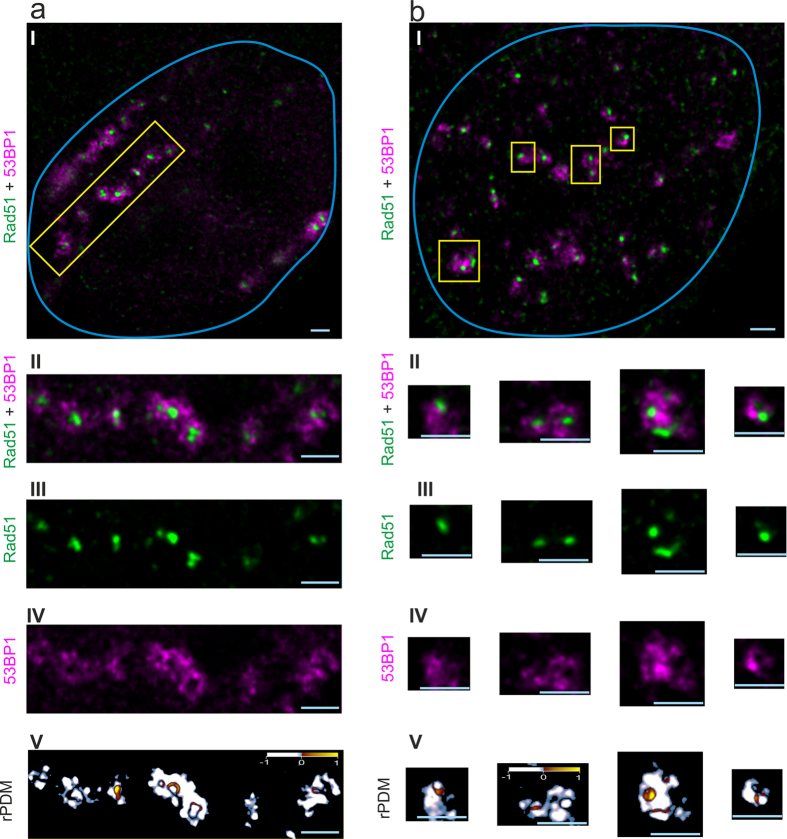
Nanoscopic images of 53BP1 and Rad51 IRIF. In (**a**), a HeLa cell nucleus (blue line), which was hit by 3 Carbon ions and in (**b**) a nucleus hit by around 200 protons are displayed. 53BP1 (magenta) and Rad51 (green) labelling visualize the DSB repair regions. Panel I shows the whole cell nuclei, whereas panels II–IV display the enlargement of the yellow-boxed repair regions. For (**a**) one ion track is enlarged and panel II shows the two-color overlay. It is visible that there are almost only magenta and green regions. This indicates that there is no correlation. The Rad51 signal shown in (**a**) panel III shows bright small spots, without any inner structure. The 53BP1 signal (**a**) panel IV appears with an inner nanostructure indicated by regions with high and regions with low intensity inside the ion track. For the cell irradiated by protons, the IRIF show anticorrelation in the overlay (**b**) panel II. The Rad51 (**b**) panel II again is visible in bright spots and the 53BP1 (**b**) panel III has again inner structure. V shows the rPDM analysis in both cases, pointing to mainly anticorrelation by showing quite exclusively white to blue regions and only few correlating red to yellow regions. Scale bar: 1 μm.

Figure 2 a shows the 53BP1 (magenta) and Rad51 (green) IRIF of a cell hit by 3 carbon ions and Fig. 2 b of a cell hit by approximately 200 protons, which corresponds to a dose of about 2 Gy. In contrast to the signal accumulation of 53BP1 and γH2AX, which occurs in all cells, Rad51 accumulation only takes place in S and G2 cells as already described^13^,^30^,^31^. Again the IRIF from proton irradiation are spread over the whole cell nucleus as already observed in Fig. 1b. In contrast the IRIF from carbon irradiation show a linear arrangement of large 53BP1 foci as already observed in Fig. 1a. Looking into the details, 53BP1 also shows round and elongated nanostructures (Fig. 2a IV, magenta channel) after carbon irradiation. The Rad51 foci (Fig. 2 III, green channel), however, consist of homogeneous, small round spots that have dimensions similar to the single nanostructures of the 53BP1. Although the macroscopic, random damage distribution for low LET proton irradiation is different to the track-like structure for carbon ion irradiation the nanostructure inside the 53BP1 IRIF is similar for both irradiation types. Beside some distinct spots the nanostructures of 53BP1 consists mainly in strings in both LET cases. Interestingly no difference in 53BP1 structure for the S/G2 cells originating from the Rad51-53BP1 experiment compared to the cell from the γH2AX-53BP1 experiment (all cell cycles) is visible. Rad51 instead shows no inner structure as visible in Fig. 2 b (green channel). The Rad51 IRIF manifests itself as small bright spots of similar sizes for both high and low LET radiation.

#### Corrrelation analysis

The reduced product of the differences from the mean (rPDM)^13^ is applied in order to obtain a quantitative, pixel-by-pixel evaluation of correlation or anticorrelation between the different repair factors. Although there is no intense γH2AX IRIF without 53BP1 accumulation and vice versa on the macro scale, correlation analysis between γH2AX and 53BP1 shows a large fraction of anticorrelating pixels within the IRIF nanostructures over the entire IRIF areas (Fig. 1 V). The rPDM image shows both positively correlating (red to yellow) and negatively correlating regions (blue to white). Dense protein accumulation in the z-direction leads to a more pronounced signal. As the resolution in z-direction is limited to 200 nm the nanostructures of γH2AX and 53BP1 cannot be completely resolved forming artificial overlap regions. Two repair factors situated in close proximity may lead to a partial overlap of the IRIF which cannot be resolved by STED nanoscopy. Thus, an even larger anticorrelation is expected if an increased resolution could be applied. This nanostructural behaviour indicates that γH2AX and 53BP1 belong to the same repair compartment but have a different localization on the nanoscale within the compartment.

By contrast, the rPDM analysis for 53BP1 and Rad51 mainly shows anticorrelation (cf. Fig. 2 V), representing the different repair compartments as already ascertained by Reindl *et al*.^13^. Due to the improved z-resolution, compared to the previous study, the correlating regions located at the border between Rad51 and 53BP1 are reduced. There are only minor correlating structures at the border between Rad51 foci and 53BP1 nanostructures being even smaller than the x- and y- resolution of 105 nm (cf. Supplementary Figure S1). Therefore, it is most probable that these small correlating regions originate from resolution rather than from biological structure, meaning that Rad51 and 53BP1 are in close proximity but still well separated.

#### Nanostructure size

**Figure 3:**
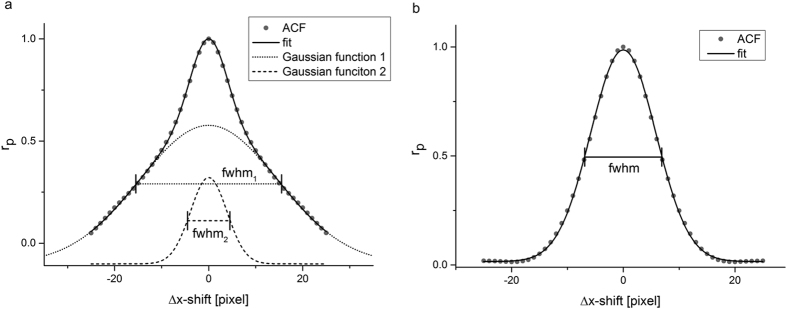
Autocorrelation fuction in measurement and fit. (**a**) Autocorrelation function of a 53BP1 IRIF at Δ*y* = 0 displayed in black. The Pearson correlation coefficient has a maximum of 1 for Δ*x* = 0 and decreases for large pixel shifts Δ*x*. A superposition of two Gaussian functions (black line) nicely fits the data (grey points). The broad Gaussian contribution (fwhm_1_) representing the IRIF gross size is plotted in the dotted line and the narrow Gaussian contribution (fwhm_2_) representing the nanostructure is plotted in the dashed line (**b**) Autocorrelation function of a Rad51 IRIF at Δ*y* = 0 displayed in grey. The data are well fitted by a single Gaussian (black line) The corresponding width is labelled as fwhm.

The size of structures repeatedly occurring in the microscope images are measured using the auto correlation function (ACF). The ACF was determined using the approach introduced by Van Steensel *et al*.^32^. Briefly, the image containing the structures of interest is duplicated and the Pearson correlation coefficient between the original image and the copy is calculated for different x- and y- displacements. For Δ*x* = 0 and Δ*y* = 0 the correlation has a maximum with Pearson coefficient of 1. The larger the shift in both directions the smaller the correlation becomes, but the decrease depends on the mean structure size in the image. The determined curves for x-shift with Δ*y* = 0 and y-shift with Δ*x* = 0 reflect the mean structure sizes as shown for an ACF-evaluation of an IRIF of a carbon irradiated cell for 53BP1 in Fig. 3a and Rad51 in Fig. 3b. The obtained ACF distributions for 53BP1 are nicely fitted by a superposition of two centred Gaussian distributions (Fig. 3a). The larger width represents the macro-structural size of the IRIF and the smaller one the nanostructure. For Rad51 only a singular Gaussian profile is obtained showing the total size of the IRIF without any nanostructure (Fig. 3b). The ACF is the analysis of the local intensity of an image, e.g. an IRIF with itself. Therefore it corresponds to a convolution of the signal with itself and it holds^33^: 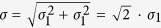. With *σ* the measured width and *σ*_1_ the actual width in the image. This is true for every structure of different size occurring in the image. The two widths of the two Gaussian functions fitted to the ACF distribution for 53BP1 and γH2AX IRIF can be interpreted as the full widths at half maximum (fwhm) of the nanostructure and the gross structure each multiplied by a factor of 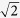.

The size measurements were done in two different labelling setups (53BP1 + Rad51, 53BP1 + γH2AX) for 2 irradiation modes on 170 IRIF altogether. The ACF analysis of γH2AX and 53BP1 show a superposition of two Gaussian in both irradiation cases, low and high LET.

The obtained full width at half maximum (fwhm) values of the IRIF are presented in Table 2. The mean gross IRIF size for 53BP1 is (433 ± 10) nm (SEM, 40 IRIF measured) for low LET proton irradiation and significantly increases with increasing LET to (540 ± 50) nm (SEM, 40 IRIF measured) for carbon ion irradiation (p < 0.05, one-sided, unpaired t-test). For γH2AX the size of (540 ± 60) nm (SEM, 20 IRIF measured) from high LET radiation is the same as for 53BP1 within the given uncertainties resulting in a pooled average size of (540 ± 50) nm. For low LET radiation the γH2AX IRIF size in the range of the 53BP1 IRIF size (390 ± 40) nm (SEM, 20 IRIF measured). The difference for high and low LET radiation is for γH2AX significant (p < 0.05, one-sided, unpaired t-test), too. The mean size (fwhm) of the Rad51 IRIF is much smaller and stays the same for low LET ((135 ± 10) nm, SEM, 20 IRIF measured) and high LET ((150 ± 14) nm, SEM, 50 IRIF measured) radiation within the given uncertainties. Remarkably, the nanostructure size for 53BP1 and γH2AX is the same as the total IRIF size for Rad51 and does practically not vary for the two irradiation conditions (high LET: (135 ± 7) nm and low LET: (122 ± 3) nm).

## Discussion

Super resolution STED nanoscopy was used to identify the structural relationships occurring in ionizing radiation induced 53BP1, γH2AX and Rad51 IRIF in human HeLa cells for low and high LET irradiation. The correlating and anticorrelating areas of IRIF from different repair factors were quantitatively analysed using the rPDM approach. By introducing the autocorrelation function as modified from the Van Steensel approach^32^ mean total IRIF sizes and the characteristic IRIF nanostructure sizes were obtained.

**Figure 4:**
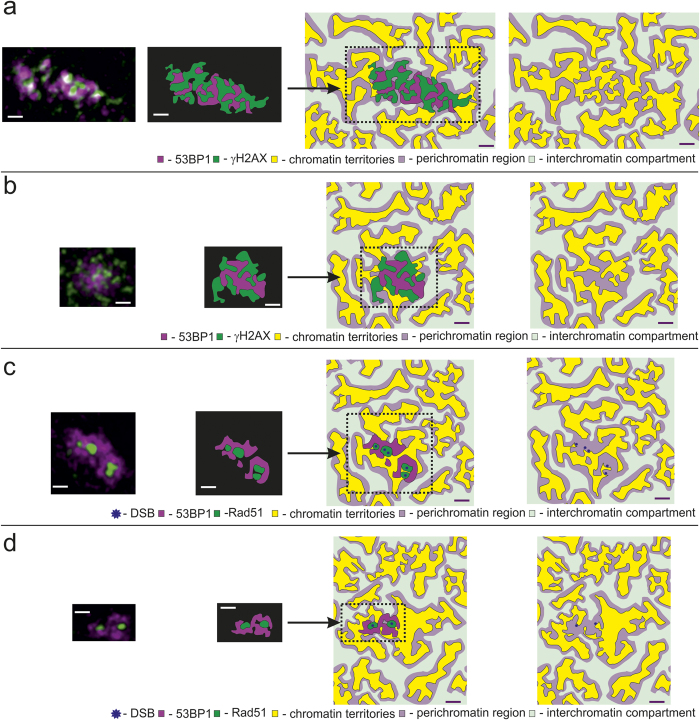
Schematic views of IRIFs according to our model. IRIF from Figs 1 and 2 are presented. First column shows the measured IRIF, the Second column the IRIF with high contrast. In the third column the IRIF are fitted into a sketch of the CT-IC model. Last column shows the sketch of the chromatin territories (yellow), the perichromatin region (light purple) and the interchromatin compartment (light green) based on our model. The chromatin/interchromatin and perichromatin regions are artificially extended to demonstrate the model. (**a**) Carbon ion irradiated γH2AX/53BP1 IRIF from Fig. 1a: γH2AX (green) is located within the chromatin compartment (yellow). 53BP1 (magenta) is part of the perichromatin region (light purple). (**b**) Proton irradiated γH2AX/53BP1 IRIF from Fig. 1b: The structure is the same as in (**a**). (**c**) Rad51/53BP1 IRIF caused by high LET radiation from Fig. 2a: Rad51 (green) is located directly around the DSB (blue star) at the border of the chromatin compartment. It is surrounded by 53BP1, which is part of the perichromatin region. (**c**) Rad51/53BP1 IRIF caused by low LET radiation from Fig. 2b: The structure is the same as in b. scale bar: 500 nm.

The microscopic structures of 53BP1 and γH2AX IRIF look quite similar: they have a similar total IRIF size of (540 ± 40) nm fwhm for carbon ion induced foci and also a same but significantly smaller IRIF size for low LET proton irradiation ((412 ± 21) nm). They also show a similar nanostructure size of about 130 nm being also the same for both, low and high LET radiation. The nanostructure size is larger than the microscope resolution (105 nm) and therefore it is concluded that this is true size of the clustered proteins. On a micrometer scale, the two repair factors correlate and due to their large IRIF sizes the conclusion can be drawn that 53BP1 and γH2AX are both part of the flanking chromatin DSB repair compartment as it has been described before^13^,^32^. However, within the nanostructure there is a partial anticorrelation between 53BP1 and γH2AX. Due to limited z-resolution, which is 200 nm in the presented experiments, most of the visible correlation between 53BP1 and γH2AX is probably artificial. Thus, a nearly complete anticorrelation between the 53BP1 and γH2AX would be expected, which is together with the associated DSB chromatin nano organization compatible with the CT-IC model^21^,^22^,^23^,^24^. γH2AX is a histone variant and therefore it may be used to mark the DNA. With this one can conclude that it labels the chromatin territory (CT). 53BP1, as DSB repair protein instead is suggested to be situated within the perichromatin region and interchromatin compartment. The schematic model is visualized in Fig. 4a and b. Due to their limited extension, the IRIF only mark a selected area of the chromatin structure in close vicinity to a radiation induced DSB. But still they properly reflect DSB domain structure, with CT, IC and perichromatin region around the DSB.

Complementary to the 53BP1 and γH2AX, the Rad51 IRIF are represented by a single structure of total size (135 ± 10) nm without any nanostructure. The Rad51 IRIF is localized in the single-stranded DNA (ssDNA) compartment^13^,^20^. When Rad51 localizes to the damage and 53BP1 is located in the surrounding regions of damage, possibly shifted apart from the DSB site by proteins like Rad51. This may lead to the anticorrelation between Rad51 and 53BP1 (see schematic drawings in Fig. 4c and d) where 53BP1 molecules remain in the surroundings of the Rad51 foci. Thus, 53BP1 and Rad51 foci colocalize when observed by fluorescence microscopy of lower resolution, i.e. confocal microscopy, as performed in the study of Ochs and co-workers^10^. The IRIF nanostructure and the relationship between Rad51 and 53BP1 do not substantially change when investigating IRIF after low LET (Fig. 4d) and high LET (Fig. 4c) irradiation. The direct attachment of the Rad51 molecules to the cleaved DNA at the DSB ends induces resection of the DNA ends while the 53BP1 in the outer regions may limit the extension of the resected strands. In conclusion, the different structural clustering of γH2AX, 53BP1 and Rad51 occurs due to their different roles throughout the DSB repair process. In addition, the measured nanostructures are in relation to chromatin structure, which might be a reason for the structure and stability of DSB repair.

In this study we used the approach of correlating the nanostructures of different repair factors to each other to get deeper insight in the higher order chromatin structure. Although no chromatin labelling was performed the results are in good correspondence to findings of former studies, where e.g. 53BP1 was found not to penetrate dense chromatin domains^12^. Whereas other proteins like NBS1 could penetrate directly to the damage independent of chromatin compaction^12^. We can conclude that our findings well agree with the models and are reasonable with the functions of the proteins through damage repair. The hypothesis that the phosphorylation of H2AX and other biological and/or physical processes help to decondens the chromatin around the damage as proposed by Falk *et al*.^12^ is reasonable. Moreover the presented work allows to connect the hypothesis of decondensation and stabilization of the damaged region by 53BP1^10^.

## Methods

### Cell-culture and irradiation

HeLa cells (AG Friedl, LMU Munich) were cultivated at 37 °C (100% humidity, 95% air +5% CO_2_) in RPMI medium (Sigma Aldrich), which was supplemented with 10% FCS and 1% Penicillin/Streptomycin. Cells were seeded on 22 × 22 *mm*^2^ coverslips several hours prior to irradiation, so that at the time of irradiation the cells were in the exponential growth phase. Irradiation was performed at the microirradiation facility SNAKE^34^ with carbon ions accelerated to 55 MeV and 21 MeV protons. During irradiation the cells were covered by a 7.5 ± 2.5 *μm* thick medium layer, which was determined by weight measurement of the sample with medium and totally dried out. The thickness was calculated as 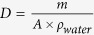, with m the mass, *A* = 22 × 22 *mm*^2^ the area of the coverslip and the density of water 
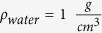
. The energy lost in the air between the beam exit nozzle and the sample, as well as in the medium covering the cells, where most of the energy is lost, led to an ion energy of (27 ± 8) MeV (LET = (500 ± 80) keV/μm) for carbon ions. The energy losses for proton irradiation were much smaller and led to an ion energy of (20.8 ± 0.1) MeV (LET = (2.6 ± 0.1) keV/μm). One minute prior to irradiation, the cover slips were taken out of the medium and dried carefully, to obtain the thickness of 7.5 ± 2.5 *μm* for the medium layer. Irradiation was performed in a field of 3.5 × 22 *mm*^2^ under an angle of 9° with respect to the cell layer and lasted a few seconds. The fluence of the carbon ion beam was 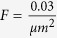 and had a variation of 20% due to ion count rate fluctuations, which lead to a dose in water 
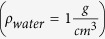
 of:


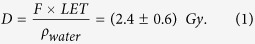


This mean dose typically resulted in two or three ion traversals per cell nucleus. Therefore, the single cell dose additionally varied by a factor of approximately 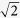, originating from Poisson statistics.

The dose for the proton beam with a fluence of 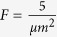 calculates to 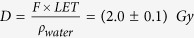. In this case, the dose spreading was much lower, because several hundred protons hit a cell and Poisson statistics does not significantly increase the error.

Dose spreading as it occurs for carbon ions in the cell nuclei is not problematic for this study because the analysis is performed on single tracks and even smaller sub-compartments and therefore on sub-nucleus level.

The factor influencing the results is the variation in DSB density, which depends on the variation of the LET, which was 16% for carbon ions and 4% for the protons.

### Antibodies and immunofluorescence detection

After 1 h of post-irradiation incubation, the cells were fixed for 15 min in a 2% (w/v) para-formaldehyde solution, washed with phosphate-buffered saline (PBS), and permeabilized with a 0.1% (v/v) Triton-X-100 solution. Then the cells were blocked with PBS containing 1% bovine serum albumin (BSA) and 0.15% glycine, as previously described^33^. The labelling was performed with mouse anti-γH2AX (m-a-γH2AX, Millipore #05-636, 1:500), rabbit anti-53BP1 (r-a-53BP1, Novus #NB100-305, 1:500) and a mouse anti-Rad51 (m-a-Rad51, GeneTex, 1:500) primary antibody and goat-anti-mouse Abberior STAR 440SXP (Abberior, 1:50) and goat-anti-rabbit Chromeo 505 (Active Motif, 1:500). There were two kinds of samples, one with m-a-γH2AX and r-a-53BP1 and the second one with m-a-Rad51 and r-a-53BP1.

### Microscopy and image analysis

A super resolution optical CW STED microscope (Leica TCS SP 8 3X) was used to obtain the detailed structures of the IRIF. For the Abberior STAR 440SX an excitation laser wavelength of 470 nm and for the Chromeo505 a wavelength of 514 nm were used. The detection ranges were 473 nm to 504 nm and 518 nm to 580 nm respectively, with a depletion laser at 592 nm. Laser power for the excitation laser is in the range of 1 mW and for the STED laser around 70 mW. The STED laser was subdivided in a lateral STED beam (40% of the energy) and an axial STED beam (60%). The system was aligned with regard to temporal and temperature dependent shift as described before^13^. Stacks of cell nuclei (3–5 μm thickness) with a slice distance of 160 nm were acquired with a 100x oil objective (Leica HCX PL APO 100x/1.4 Oil) and a pixel size of 40 nm. The raw data images were processed using spectral unmixing and deconvoluted using Huygens professional (Scientific Volume Imaging) as described before^13^, resulting in a lateral resolution of 105 nm and an axial resolution of 200 nm.

### Sample size determination

The measurements were performed in 3 different experiments which were carried out twice as displayed in Table 1. The standard deviation within one experiment is approx. *σ* = 50*nm* for the nanostructure and approx. *σ* = 180 *nm*. The sample size is calculated as 
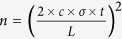
, with n sample size, L the width of the confidence interval, the t-factor and c-factor^35^. For small sample sizes the t-factor has to be used. For a sample size of 10 the t factor is t = 1.053. For the same sample size a confidence interval of 90% leads to a c-factor of c = 1.645^35^. For the nanostructure the width of the confidence interval is L = 60 nm and the sample size calculates to n = 9. For the IRIF size L = 200 nm and the sample size is n = 10. Thus the smallest possible sample size for each experiment is n = 10. For carbon ion irradiation the number of Rad51 IRIF is larger, because there are several Rad51 IRIF within one 53BP1 IRIF. After the second repetition the results were compared and pooled, since the variation within one experiment was larger than in between the experiments. For the proton experiment γH2AX + 53BP1 only one repetition was performed. To enable a comparison of the results of the pooled experiments and this single experiment 20 IRIF were taken for this experiment.

### Data analysis

#### Correlation measurement using the reduced product of the differences from the mean (rPDM)

For correlation analysis, the rPDM method as described by Reindl *et al*.^13^ was used. Briefly, for each IRIF the mean intensities in both channels are calculated. After this, pixels are expulsed where both pixel values are lower than the mean. These correlations are not considered due to the lack of protein present. For each residual pixel the normalized product of the differences from the mean


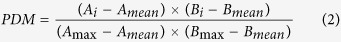


is calculated. The value ranges from −1 for anticorrelation to +1 for correlation. A 2D Correlation map is formed where the individual pixel correlation coefficient is reprinted as an image.

#### Size measurement with the Van Steensel approach

The samples were analyzed using cross correlation functions (CCF) as introduced by Van Steensel *et al*.^32^ expanded in two dimensions as a basis to obtain the size of the detailed nanostructure occurring inside the IRIF. The Van Steensel approach is a powerful tool to obtain the cross-correlation function (CCF) in x-direction of two images by shifting one image over a distance Δx over the other image, and calculating the Pearson correlation coefficient r for each shift, which is defined according to:


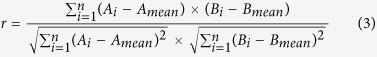


with *A*_*i*_, *B*_*i*_ the gray values for the pixel i in the first and second image, respectively. *A*_*mean*_, *B*_*mean*_ are the mean gray values for each image. The CCF is then obtained by plotting the Pearson coefficient against the shift Δ*x*. For the determination of the structure sizes of an IRIF originating from one damage marker, i.e. γH2AX, 53BP1 or Rad51, it is necessary to obtain the so called autocorrelation function (ACF), which is the CCF for an image correlated with itself. In this study, the shifts were performed in x- as well as in y-direction, which creates a shifting matrix where the columns represent the x-shift, the rows the y-shift and the entries are the Pearson correlation coefficient r for the corresponding x- and y-shift. By plotting the Pearson coefficient against both x- and y-shift, a 2D CCF or ACF can be determined. For the quantitative analysis the projection of the 2D CCF/ACF both for x- shift (Δ*y* = 0) and for y-shift (Δ*y* = 0) are used.

The ACF is a symmetrical correlation problem and therefore the ACF reduces to a convolution problem^36^. In this analysis, the underlying structures are assumed as Gaussian shaped structures for simplification. The convolution of two Gaussian functions is again a Gaussian function, where the width is 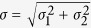
33. Convoluting a Gaussian function with itself, the resulting width is the width of the underlying Gaussian multiplied by a factor of 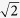.

In an image, which has several structures, like micrometer and nanostructure, the structure can be represented by a sum of several Gaussian functions. Therefore, for determining the structure sizes, a superposition of several Gaussian functions was fitted to the projection of the 2D ACF. The resulting full width at half maximum (fwhm) of each Gaussian represents one underlying structure size multiplied by the factor 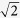. The number of Gaussian curves depends on the number of underlying structures, which have to be larger than the resolution. If there are too many Gaussian functions assumed in the fit, the FWHM or the corresponding amplitude drop to 0.

In the case of the damage markers used in this study, one or two structure sizes are visible, respectively, the size of the IRIF and the nanostructure size. They are determined using a superposition of two Gaussian functions:


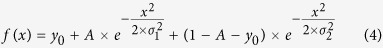


*y*_0_ is the mean Pearson correlation coefficient for large shifts, A is the amplitude of one of the two Gaussian functions and, as the maximum occurring value is 1, the height of the second Gaussian is (1−*A*−*y*_0_)). *σ*_1_ and *σ*_2_ are the standard deviations of the two Gaussian functions. Using the standard deviations the fwhm can be calculated to *fwhm*_2_ = 2.35 × *σ*_2_ and *fwhm*_1_ = 2.35 × *σ*_1_.

The sum of the Gaussians f(x) is suitable for the ACF either for γH2AX or 53BP1 where the first Gaussian function describes the IRIF macrostructure with fwhm_1_ representing the size of the IRIF. The second Gaussian function describes the smaller structure occurring inside an IRIF with a size of fwhm_2_. For Rad51 the second structure size drops to 0, because no visible nanostructure occurred and only the size determined by fwhm_1_ remains.

## Additional Information

**How to cite this article**: Reindl, J. *et al*. Chromatin organization revealed by nanostructure of irradiation induced γH2AX, 53BP1 and Rad51 foci. *Sci. Rep.* **7**, 40616; doi: 10.1038/srep40616 (2017).

**Publisher's note:** Springer Nature remains neutral with regard to jurisdictional claims in published maps and institutional affiliations.

## Supplementary Material

Supplementary Information

## Figures and Tables

**Table 1:** Experiment plan.

Experiment	Irradiation	Proteins	Sample size	Repetition
1	Carbon ions	γH2AX + 53BP1	10	2
2	Carbon ions	Rad51 + 53BP1	25 (Rad51) + 10 (53BP1)	2
3	Protons	Rad51 + 53BP1	10 (Rad51) + 10 (53BP1)	2
4	Protons	γH2AX + 53BP1	19 (γH2AX) + 20 (53BP1)	1

**Table 2:** Total IRIF sizes and the sizes of their nanostructure (fwhm values) of the different repair factors.

Protein	Carbon ion irradiation			Proton irradiation		
	IRIF size [nm]	Nanostructure size [nm]	# of analyzed IRIF	IRIF size [nm]	Nanostructure size [nm]	# of analyzed IRIF
53BP1	540 ± 50	135 ± 7	40	433 ± 10	125 ± 2	40
γH2AX	540 ± 60	135 ± 7	20	390 ± 40	119 ± 4	19
Rad51	150 ± 14	—	50	135 ± 10	—	20

The uncertainties are given as the standard error of the mean (SEM).
