# Supplementary material for: Chromatin organization revealed by nanostructure of irradiation induced γH2AX, 53BP1 and Rad51 foci
Source: Sci Rep. 2017 Jan 17;7:40616. doi: 10.1038/srep40616 (PMC5240115; doi:10.1038/srep40616)
Supplement: Supplementary Information [file srep40616-s1.pdf]

Manuscript title: "Chromatin organization revealed by nanostructure of irradiation induced γH2AX, 53BP1 and Rad51 foci"

Judith Reindl, Stefanie Girst, Dietrich W.M. Walsh , Christoph Greubel, Benjamin Schwarz,

Christian Siebenwirth, Guido A. Drexler, Anna A. Friedl, Günther Dollinger

Supplementary material

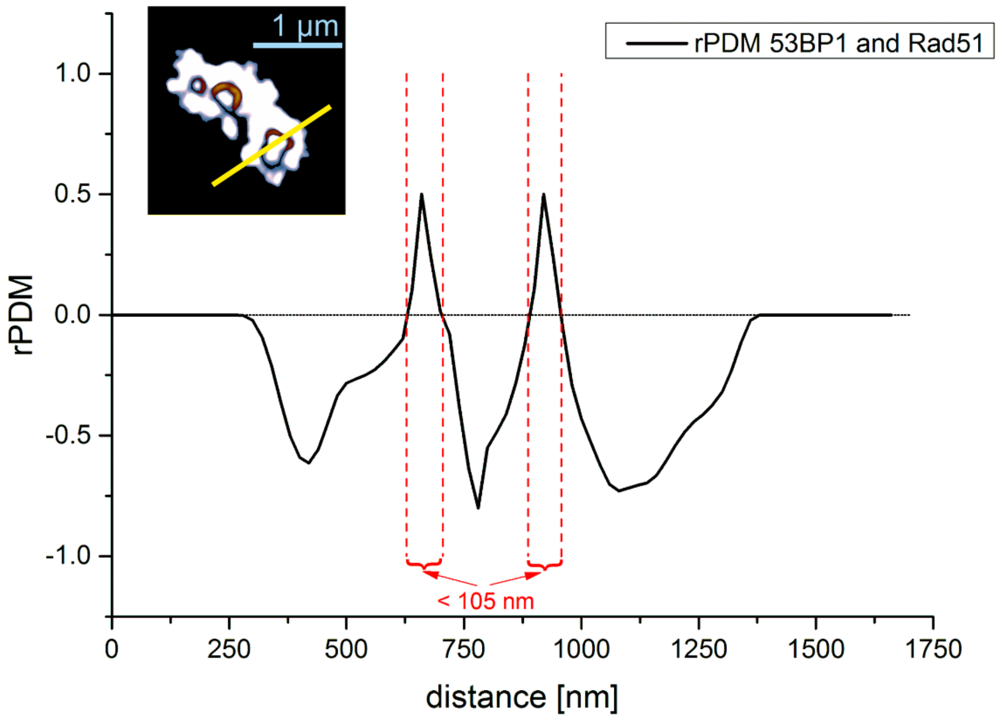

Supplementary Figure S 1: Overlap in correlation smaller than resolution The rPDM value is plotted for an 53BP1 and Rad51 IRIF along the yellow line. The positive correlating regions (rPDM>1) are smaller than the 105 nm resolution.
